# Supplementary material for: Deep multiomics profiling of brain tumors identifies signaling networks downstream of cancer driver genes
Source: Nat Commun. 2019 Aug 16;10:3718. doi: 10.1038/s41467-019-11661-4 (PMC6697699; doi:10.1038/s41467-019-11661-4)
Supplement: Supplementary file 1 — Supplementary Information [file 41467_2019_11661_MOESM1_ESM.pdf]

Deep Multiomics Profiling of Brain Tumors Identifies Signaling Networks Downstream of Cancer Driver Genes

Wang et al

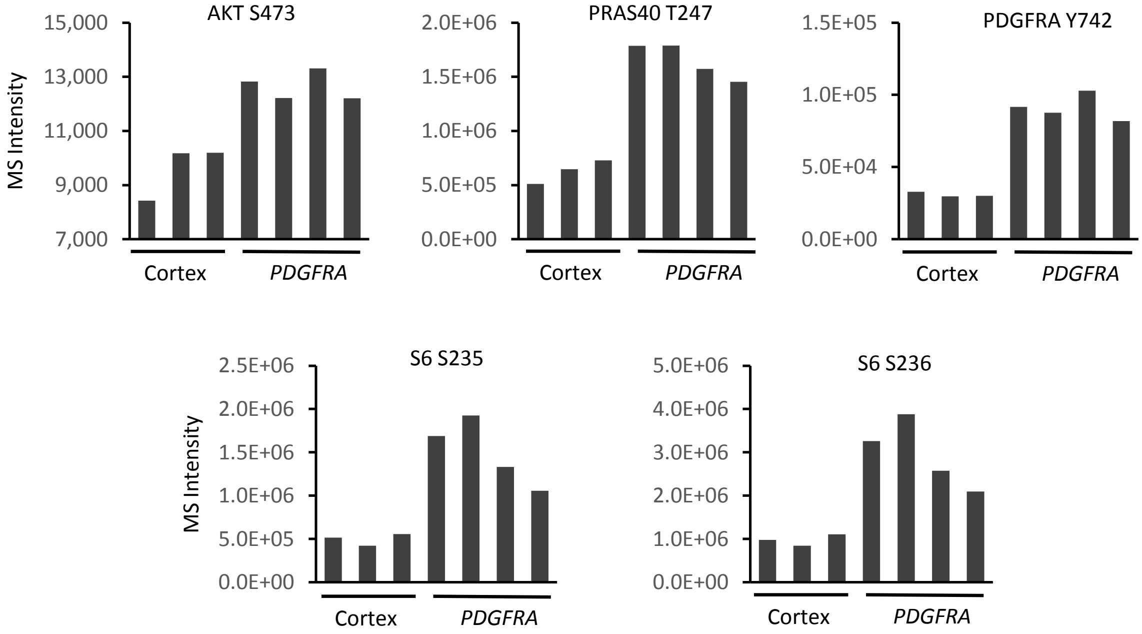

Supplementary Figure 1 | MS-based quantification is accurate

MS measurements of phosphorylation events are highly consistent with previous immunoblot assays comparing *PDGFRA*-driven HGGs to normal cortex (Paugh BS *et al*. 2013).

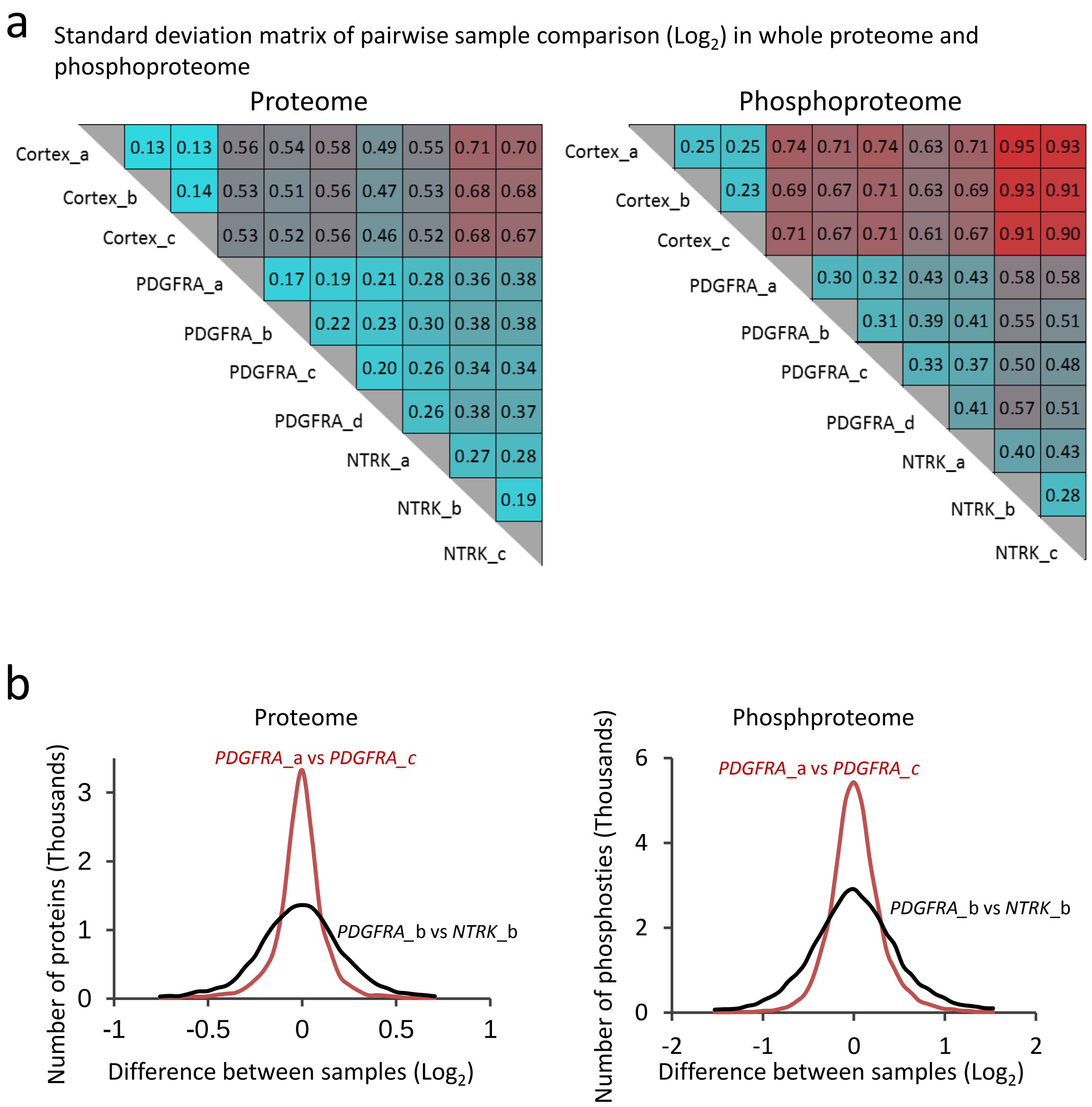
a
Standard deviation matrix of pairwise sample comparison (Log2) in whole proteome and phosphoproteome
Proteome
Cortex_a 0.13 0.13 0.56 0.54 0.58 0.49 0.55 0.71 0.70
Cortex_b 0.14 0.53 0.51 0.56 0.47 0.53 0.68 0.68
Cortex_c 0.53 0.52 0.56 0.46 0.52 0.68 0.67
PDGFRA_a 0.17 0.19 0.21 0.28 0.36 0.38
PDGFRA_b 0.22 0.23 0.30 0.38 0.38
PDGFRA_c 0.20 0.26 0.34 0.34
PDGFRA_d 0.26 0.38 0.37
NTRK_a 0.27 0.28
NTRK_b 0.19
NTRK_c
Phosphoproteome
Cortex_a 0.25 0.25 0.74 0.71 0.74 0.63 0.71 0.95 0.93
Cortex_b 0.23 0.69 0.67 0.71 0.63 0.69 0.93 0.91
Cortex_c 0.71 0.67 0.71 0.61 0.67 0.91 0.90
PDGFRA_a 0.30 0.32 0.43 0.43 0.58 0.58
PDGFRA_b 0.31 0.39 0.41 0.55 0.51
PDGFRA_c 0.33 0.37 0.50 0.48
PDGFRA_d 0.41 0.57 0.51
NTRK_a 0.40 0.43
NTRK_b 0.28
NTRK_c
b
Proteome
Number of proteins (Thousands)
PDGFRA_a vs PDGFRA_c
PDGFRA_b vs NTRK_b
0
1
2
3
-1
-0.5
0
0.5
1
Difference between samples (Log2)
Phosphproteome
Number of phosphosties (Thousands)
PDGFRA_a vs PDGFRA_c
PDGFRA_b vs NTRK_b
0
2
4
6
-2
-1
0
1
2
Difference between samples (Log2)

Supplementary Figure 2 | MS-based proteomic analyses specify particularly small variations

(a) Pairwise standard deviation matrix of whole proteome and phosphoproteome displays particularly small variations between replicates.

(b) Examples show distributions of representative Log_2 level whole proteome and phosphoproteome variations between *PDGFRA*-driven HGG biological replicates and between two HGG tumors.

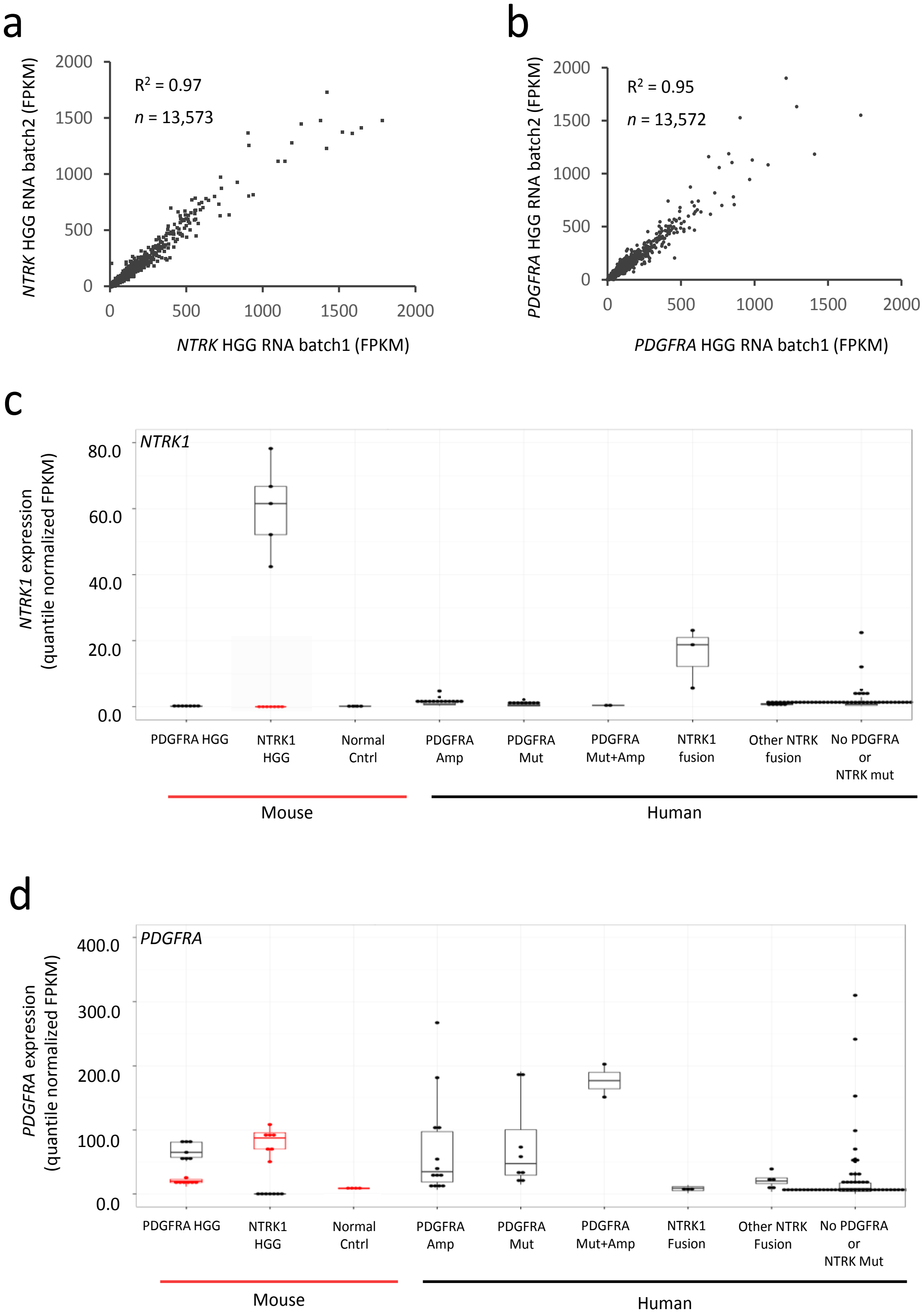
a
R^2 = 0.97
n = 13,573
NTRK HGG RNA batch2 (FPKM)
NTRK HGG RNA batch1 (FPKM)
b
R^2 = 0.95
n = 13,572
PDGFRA HGG RNA batch2 (FPKM)
PDGFRA HGG RNA batch1 (FPKM)
c
NTRK1
NTRK1 expression (quantile normalized FPKM)
PDGFRA HGG
NTRK1 HGG
Normal Cntrl
PDGFRA Amp
PDGFRA Mut
PDGFRA Mut+Amp
NTRK1 fusion
Other NTRK fusion
No PDGFRA or NTRK mut
Mouse
Human
d
PDGFRA
PDGFRA expression (quantile normalized FPKM)
PDGFRA HGG
NTRK1 HGG
Normal Cntrl
PDGFRA Amp
PDGFRA Mut
PDGFRA Mut+Amp
NTRK1 Fusion
Other NTRK Fusion
No PDGFRA or NTRK Mut
Mouse
Human

Supplementary Figure 3 | HGG mouse models are reproducible

(a, b) Comparisons of RNAseq results on batch2 and batch1 HGG mouse models show highly consistent transcript abundance from tumors generated by independent experiments. Scatterplots show FPKM values of genes in batch1 and batch2 HGG mouse models, Pearson correlations were performed and R^2 values were shown on the upper left side of the panels.

(c, d) Mouse tumors express levels of mutated PDGFRA or NTRK fusion genes that are relevant to the levels expressed in primary human tumors. Normalized RNAseq data was used to compare the expression of the human NTRK1 (c) or PDGFRA (d) expression in mouse HGGs, control mouse cortex, or human HGGs from Wu et al, Nat Genet, 2014, categorized as HGGs carrying amplified wild-type PDGFRA, mutated PDGFRA, amplified and mutated PDGFRA, TPM3-TRK1 fusion gene, fusion genes involving NTRK2 or 3, or HGGs without mutation in PDGFRA or NTRK genes. Expression of mouse orthologs for NTRK1 (c) or PDGFRA (d) are shown in red. Analysis of variant allele frequency from whole genome sequencing data showed that human tumors with TPM3-NTRK1 fusion gene carried the fusion gene as a subclonal population comprising only 9-20% of the tumor cells. Therefore, expression of the TPM3-NTRK1 from bulk tumor RNA is a significant underestimate of expression of the TPM3-NTRK1 fusion in human tumor cells. Boxplot center line, median; box limits, upper and lower quartiles; whiskers, 1.5x interquartile range; points, outliers.

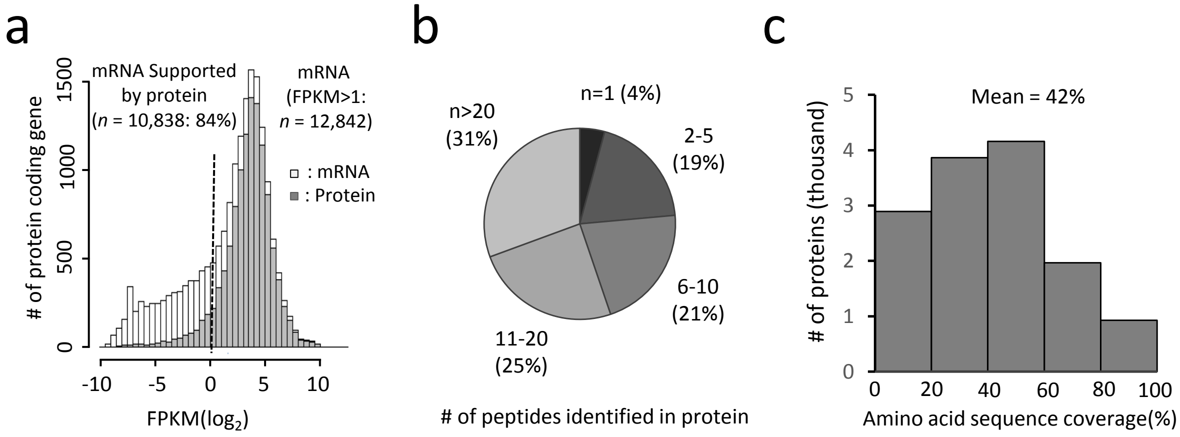

Supplementary Figure 4 | Proteome-transcriptome comparison shows deep proteomic coverage

(a) High percentage (84%) of expressed transcripts detected by MS. In the histogram of transcript log_2FPKM, MS-detected proteins are highlighted in grey. To ensure data stringency, FPKM >1 were applied in this analysis.

(b) The number of peptides identified for each protein in this MS analysis. > 96% of proteins were identified by at least 2 distinct peptides.

(c) The MS coverage of theoretically observable amino acid sequences in a histogram. In average, 42% of observable amino acid sequences were detected in this MS study.

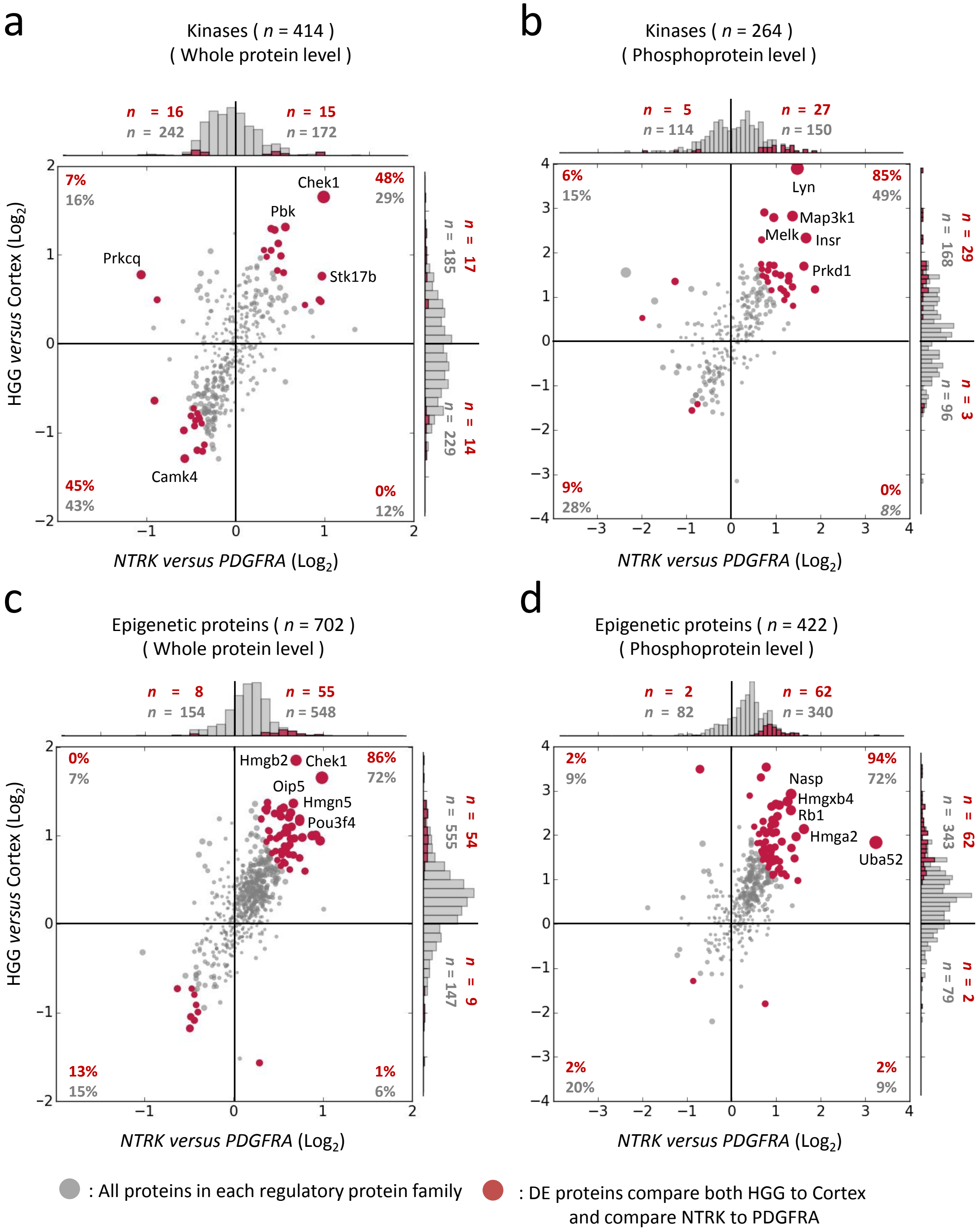
a
Kinases (n = 414)
(Whole protein level)
b
Kinases (n = 264)
(Phosphoprotein level)
c
Epigenetic proteins (n = 702)
(Whole protein level)
d
Epigenetic proteins (n = 422)
(Phosphoprotein level)
HGG versus Cortex (Log2)
NTRK versus PDGFRA (Log2)
Chek1
Pbk
Prkcq
Stk17b
Camk4
Lyn
Map3k1
Melk
Insr
Prkd1
Hmgb2
Chek1
Oip5
Hmgn5
Pou3f4
Nasp
Hmgxb4
Rb1
Hmga2
Uba52
: All proteins in each regulatory protein family
: DE proteins compare both HGG to Cortex and compare NTRK to PDGFRA

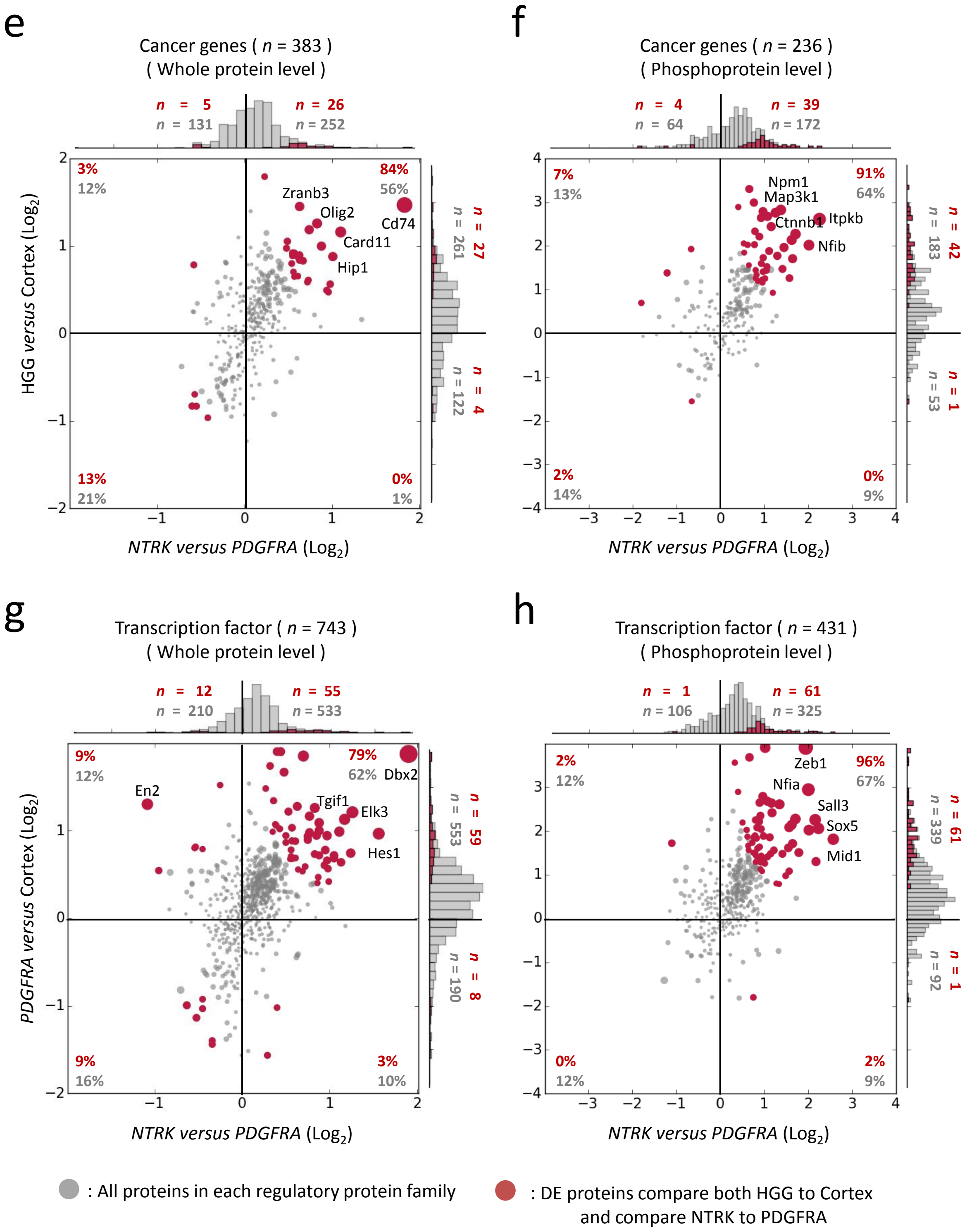
e
Cancer genes (n = 383)
(Whole protein level)
n = 5
n = 131
n = 26
n = 252
3%
12%
84%
56%
13%
21%
0%
1%
Zranb3
Olig2
Cd74
Card11
Hip1
n = 27
n = 261
n = 4
n = 122
HGG versus Cortex (Log2)
NTRK versus PDGFRA (Log2)
f
Cancer genes (n = 236)
(Phosphoprotein level)
n = 4
n = 64
n = 39
n = 172
7%
13%
91%
64%
2%
14%
0%
9%
Npm1
Map3k1
Ctnnb1
Itpkb
Nfib
n = 42
n = 183
n = 1
n = 53
NTRK versus PDGFRA (Log2)
g
Transcription factor (n = 743)
(Whole protein level)
n = 12
n = 210
n = 55
n = 533
9%
12%
79%
62%
9%
16%
3%
10%
En2
Dbx2
Tgif1
Elk3
Hes1
n = 59
n = 553
n = 8
n = 190
PDGFRA versus Cortex (Log2)
NTRK versus PDGFRA (Log2)
h
Transcription factor (n = 431)
(Phosphoprotein level)
n = 1
n = 106
n = 61
n = 325
2%
12%
96%
67%
0%
12%
2%
9%
Zeb1
Nfia
Sall3
Sox5
Mid1
n = 61
n = 339
n = 1
n = 92
NTRK versus PDGFRA (Log2)
: All proteins in each regulatory protein family
: DE proteins compare both HGG to Cortex and compare NTRK to PDGFRA

Supplementary Figure 5 | Proteomic analyses detect a global trend of increased expression and phosphorylation of regulatory protein families in HGG tumors

(a - h) Deep proteomic data analyses show a global increase of protein expression and phosphorylation of most of regulatory protein families (Kinase, epigenetic genes, transcription factors and cancer genes) in HGG tumors compare to cortex, with higher magnitude of increase in *NTRK*-driven HGG than *PDGFRA*-driven HGG. Scatter-histogram graphs of regulatory family proteins expression and phosphorylation comparing both HGG tumors to cortex and *NTRK*-driven HGGs to *PDGFRA*-driven HGGs. B.H. adjusted Student T test *p* values of 0.05 in both pairwise comparisons plus fold change (FC) cutoff of $FC_{(NTRK/PDGFRA)} * FC_{(HGG/cortex)} > 1.5^2$ for proteome and >2 for phosphoproteome are applied for differential expression analyses. DE genes are shown in red. Magnitude of change is represented by dot size. Top five altered proteins and phosphoproteins with the largest magnitude of alterations are labeled. The distributions of pairwise differences are shown by histograms.

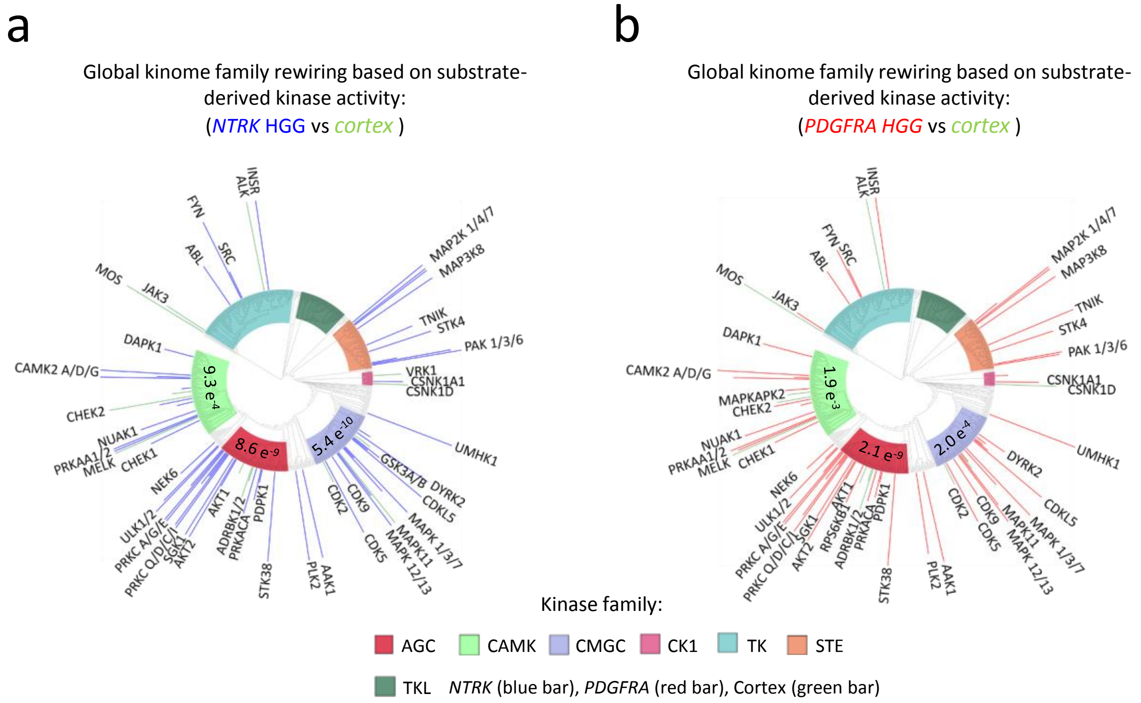
a
Global kinome family rewiring based on substrate-derived kinase activity:
(NTRK HGG vs cortex)
9.3 e-4
8.6 e-9
5.4 e-10
b
Global kinome family rewiring based on substrate-derived kinase activity:
(PDGFRA HGG vs cortex)
1.9 e-3
2.1 e-9
2.0 e-4
Kinase family:
AGC
CAMK
CMGC
CK1
TK
STE
TKL
NTRK (blue bar), PDGFRA (red bar), Cortex (green bar)

Supplementary Figure 6 | AGC, CAMK and CMGC kinase superfamilies display higher activity in both HGG tumors compare to cortex

(a) Kinome tree circular map shows kinase activity comparing *NTRK*-driven HGG to cortex. Pairwise comparisons of kinase superfamilies with a Chi-square *P* values < 0.05 are labelled. Magnitude of kinase activity difference is represented by length of bars located outside of the kinome tree circles. Blue bar indicates higher activity in *NTRK*-driven HGG compare to Cortex. Green bar indicates higher activity in cortex compare to *NTRK*-driven HGG

(b) Kinome tree circular map shows kinase activity comparing *PDGFRA*-driven HGG to cortex. Pairwise comparisons of kinase superfamilies with a Chi-square *P* values < 0.05 are labelled. Magnitude of kinase activity difference is represented by length of bars located outside of the kinome tree circles. Red bar indicates higher activity in *PDGFRA*-driven HGG compare to Cortex. Green bar indicates higher activity in cortex compare to *PDGFRA*-driven HGG

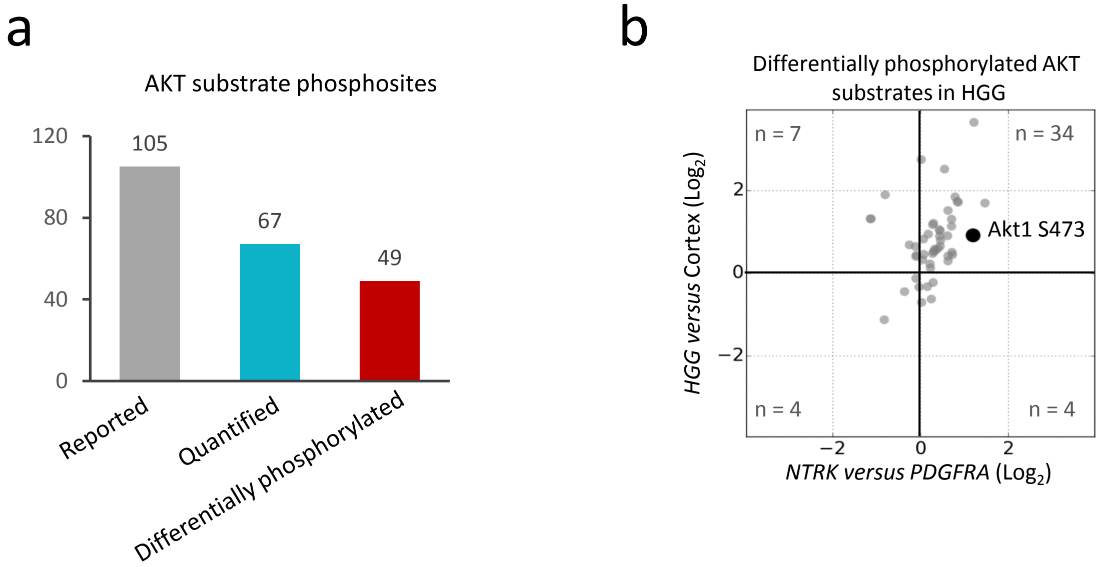

Supplementary Figure 7| Evaluation of AKT regulated substrates

(a) A high percentage of known AKT substrate sites were identified in the deep phosphoproteome. Bar graph shows reported substrate sites, quantified phosphosites and differentially phosphorylated sites.

(b) Differentially phosphorylated substrates that are regulated by AKT in HGGs. Scatter plot of $\log_2$ level changes compare HGG tumors to cortex and compare *NTRK*-driven HGG to *PDGFRA*-driven HGG. Substrates with the same phosphorylation pattern as the AKT active site (S473, located at upper right) were accepted as AKT regulated substrates.

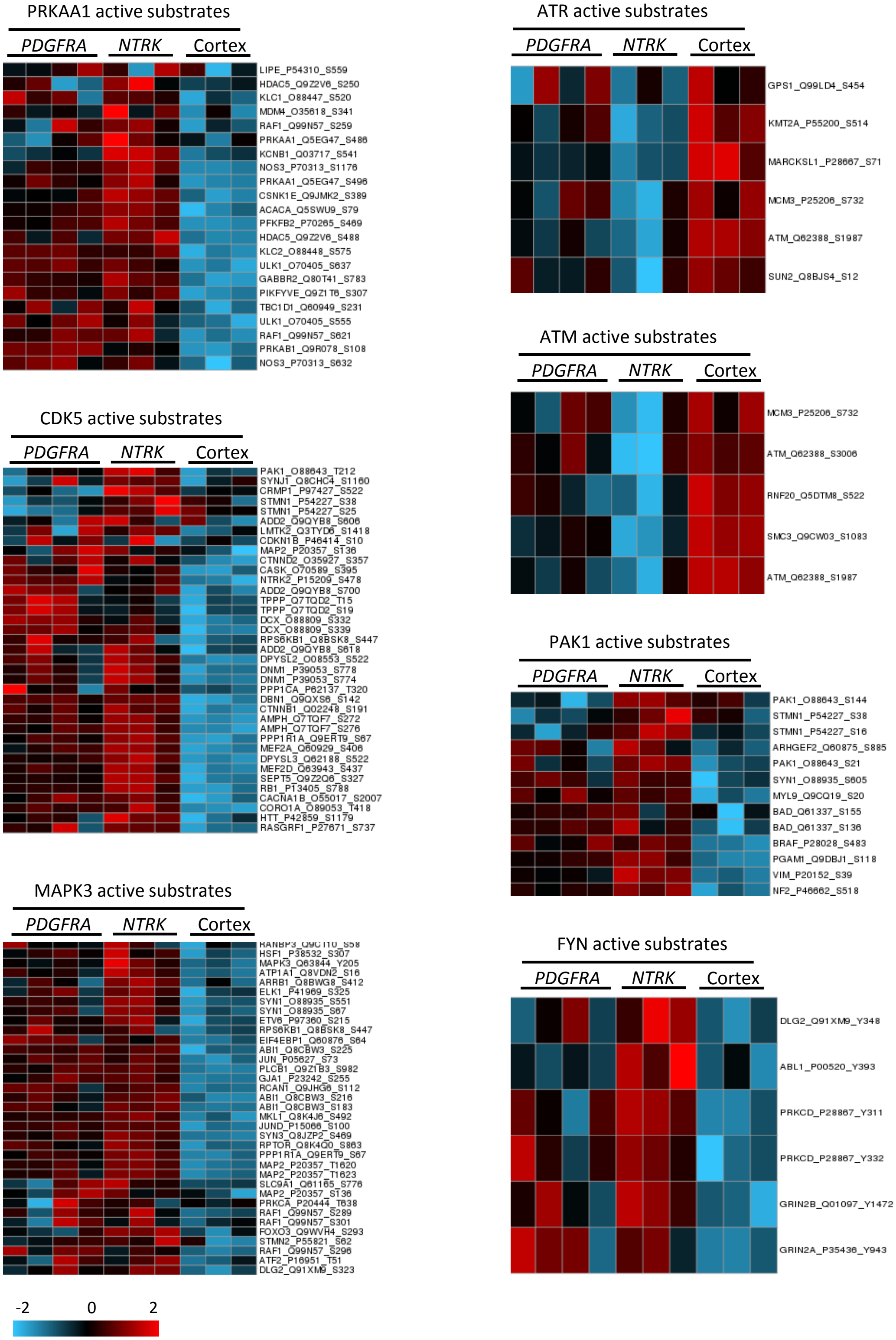
PRKAA1 active substrates
PDGFRA
NTRK
Cortex
LIPE_P54310_S559
HDAC5_Q9Z2V6_S250
KLC1_O88447_S520
MDM4_O35618_S341
RAF1_Q99N57_S259
PRKAA1_Q5EG47_S486
KCNB1_Q03717_S541
NOS3_P70313_S1176
PRKAA1_Q5EG47_S496
CSNK1E_Q9JMK2_S389
ACACA_Q5SWU9_S79
PFKFB2_P70265_S469
HDAC5_Q9Z2V6_S488
KLC2_O88448_S575
ULK1_O70405_S637
GABBR2_Q80T41_S783
PIKFYVE_Q9Z1T6_S307
TBC1D1_Q60949_S231
ULK1_O70405_S555
RAF1_Q99N57_S621
PRKAB1_Q9R078_S108
NOS3_P70313_S632
CDK5 active substrates
PDGFRA
NTRK
Cortex
PAK1_O88643_T212
SYNJ1_Q8CHC4_S1160
CRMP1_P97427_S522
STMN1_P54227_S38
STMN1_P54227_S25
ADD2_Q9QYB8_S606
LMTK2_Q3TYD6_S1418
CDKN1B_P46414_S10
MAP2_P20357_S136
CTNND2_O35927_S357
CASK_O70589_S395
NTRK2_P15209_S478
ADD2_Q9QYB8_S700
TPPP_Q7TQD2_T15
TPPP_Q7TQD2_S19
DCX_O88809_S332
DCX_O88809_S339
RPS6KB1_Q8BSK8_S447
ADD2_Q9QYB8_S618
DPYSL2_O08553_S522
DNM1_P39053_S778
DNM1_P39053_S774
PPP1CA_P62137_T320
DBN1_Q9QXS6_S142
CTNNB1_Q02248_S191
AMPH_Q7TQF7_S272
AMPH_Q7TQF7_S276
PPP1R1A_Q9ERT9_S67
MEF2A_Q60929_S406
DPYSL3_Q62188_S522
MEF2D_Q63943_S437
SEPT5_Q9Z2Q6_S327
RB1_P13405_S788
CACNA1B_O55017_S2007
CORO1A_O89053_T418
HTT_P42859_S1179
RASGRF1_P27671_S737
MAPK3 active substrates
PDGFRA
NTRK
Cortex
RANBP3_Q9CT10_S58
HSF1_P38532_S307
MAPK3_Q63844_Y205
ATP1A1_Q8VDN2_S16
ARRB1_Q8BWG8_S412
ELK1_P41969_S325
SYN1_O88935_S551
SYN1_O88935_S67
ETV6_P97360_S215
RPS6KB1_Q8BSK8_S447
EIF4EBP1_Q60876_S64
ABI1_Q8CBW3_S225
JUN_P05627_S73
PLCB1_Q9Z1B3_S982
GJA1_P23242_S255
RCAN1_Q9JHG6_S112
ABI1_Q8CBW3_S216
ABI1_Q8CBW3_S183
MKL1_Q8K4J6_S492
JUND_P15066_S100
SYN3_Q8JZP2_S469
RPTOR_Q8K4Q0_S863
PPP1R1A_Q9ERT9_S67
MAP2_P20357_T1620
MAP2_P20357_T1623
SLC9A1_Q61165_S776
MAP2_P20357_S136
PRKCA_P20444_T638
RAF1_Q99N57_S289
RAF1_Q99N57_S301
FOXO3_Q9WVH4_S293
STMN2_P55821_S62
RAF1_Q99N57_S296
ATF2_P16951_T51
DLG2_Q91XM9_S323
-2
0
2
ATR active substrates
PDGFRA
NTRK
Cortex
GPS1_Q99LD4_S454
KMT2A_P55200_S514
MARCKSL1_P28667_S71
MCM3_P25206_S732
ATM_Q62388_S1987
SUN2_Q8BJS4_S12
ATM active substrates
PDGFRA
NTRK
Cortex
MCM3_P25206_S732
ATM_Q62388_S3006
RNF20_Q5DTM8_S522
SMC3_Q9CW03_S1083
ATM_Q62388_S1987
PAK1 active substrates
PDGFRA
NTRK
Cortex
PAK1_O88643_S144
STMN1_P54227_S38
STMN1_P54227_S16
ARHGEF2_Q60875_S885
PAK1_O88643_S21
SYN1_O88935_S605
MYL9_Q9CQ19_S20
BAD_Q61337_S155
BAD_Q61337_S136
BRAF_P28028_S483
PGAM1_Q9DBJ1_S118
VIM_P20152_S39
NF2_P46662_S518
FYN active substrates
PDGFRA
NTRK
Cortex
DLG2_Q91XM9_Y348
ABL1_P00520_Y393
PRKCD_P28867_Y311
PRKCD_P28867_Y332
GRIN2B_Q01097_Y1472
GRIN2A_P35436_Y943

Supplementary Figure 8 | Heatmaps display differentially phosphorylated substrates (with up-regulated phosphorylation in HGG tumors) of other active kinases derived from kinase-substrate analysis. Color key presents the Z score of kinase substrates

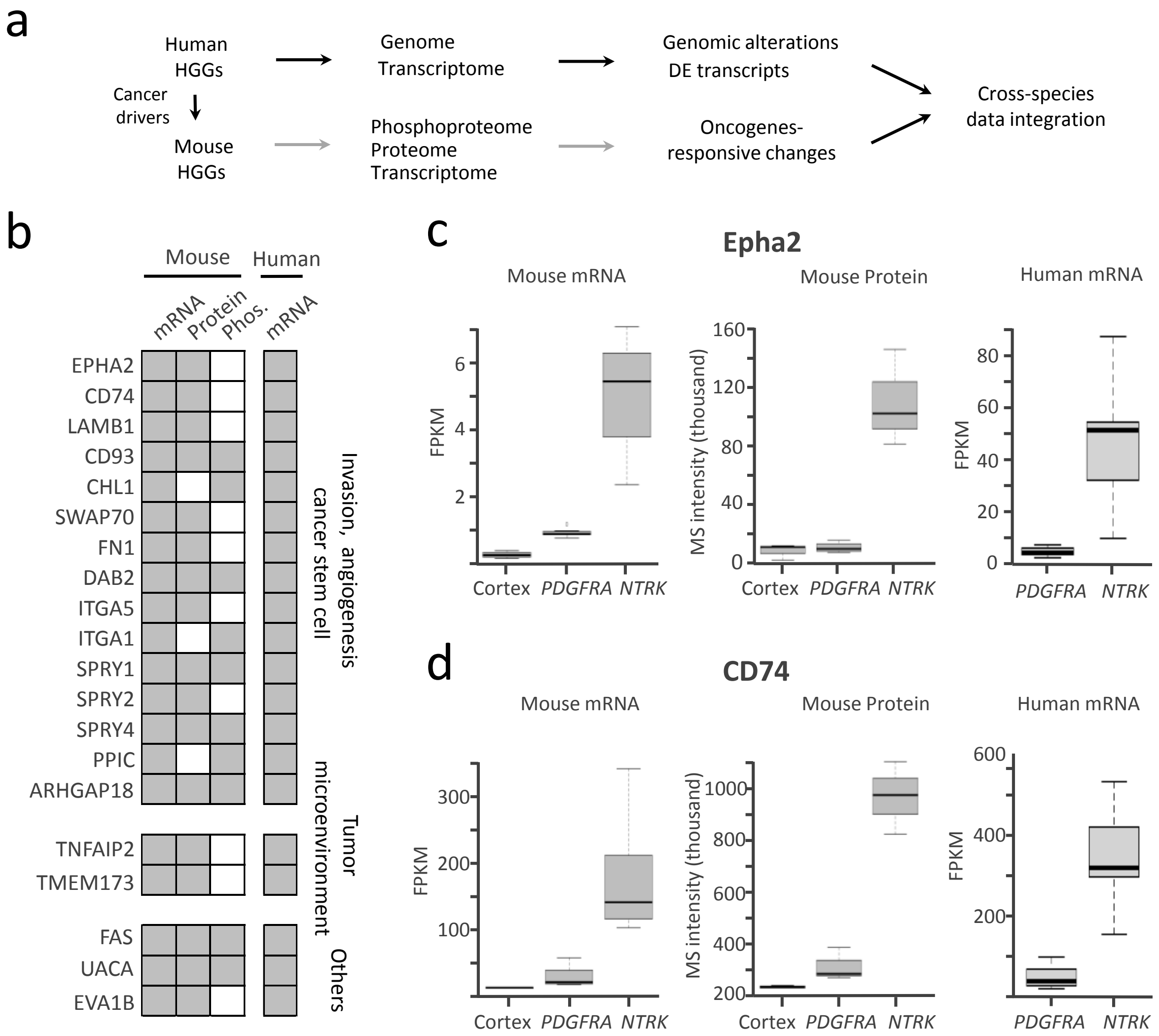
a
Human HGGs
Cancer drivers
Mouse HGGs
Genome
Transcriptome
Phosphoproteome
Proteome
Transcriptome
Genomic alterations
DE transcripts
Oncogenes-responsive changes
Cross-species data integration
b
Mouse
Human
mRNA
Protein
Phos.
mRNA
EPHA2
CD74
LAMB1
CD93
CHL1
SWAP70
FN1
DAB2
ITGA5
ITGA1
SPRY1
SPRY2
SPRY4
PPIC
ARHGAP18
TNFAIP2
TMEM173
FAS
UACA
EVA1B
Invasion, angiogenesis cancer stem cell
Tumor microenvironment
Others
c
Epha2
Mouse mRNA
Mouse Protein
Human mRNA
FPKM
MS intensity (thousand)
Cortex
PDGFRA
NTRK
d
CD74
Mouse mRNA
Mouse Protein
Human mRNA
FPKM
MS intensity (thousand)
Cortex
PDGFRA
NTRK

Supplementary Figure 9 | Combination of mouse and human HGG data prioritizes putative cancer genes.

(a) Overview of multiomics analysis across species

(b) Cross species integrative analysis reveal cancer driver responsive genes, many of which are well-reported cancer genes in HGG. The cancer driver responsive genes were classified according to their functions. Mouse transcripts and proteins/phosphorylations that follow the expression order of *NTRK* > *PDGFRA* > Cortex were accepted to be oncogene responsive. Human transcripts that show *NTRK* > *PDGFRA* were intersected with mouse oncogene responsive genes for prioritization. Gray fills indicate consistent changes identified, while white boxes indicate no significant change or data not available.

(c, d) Boxplots show Epha2 and CD74 expression in multiomics data in human and mouse. Human mRNA boxplots compare expression levels of EPHA2 or CD74 in pediatric HGGs with mutated PDGFRA or NTRK fusion genes. Boxplot center line, median; box limits, upper and lower quartiles; whiskers, 1.5x interquartile range.

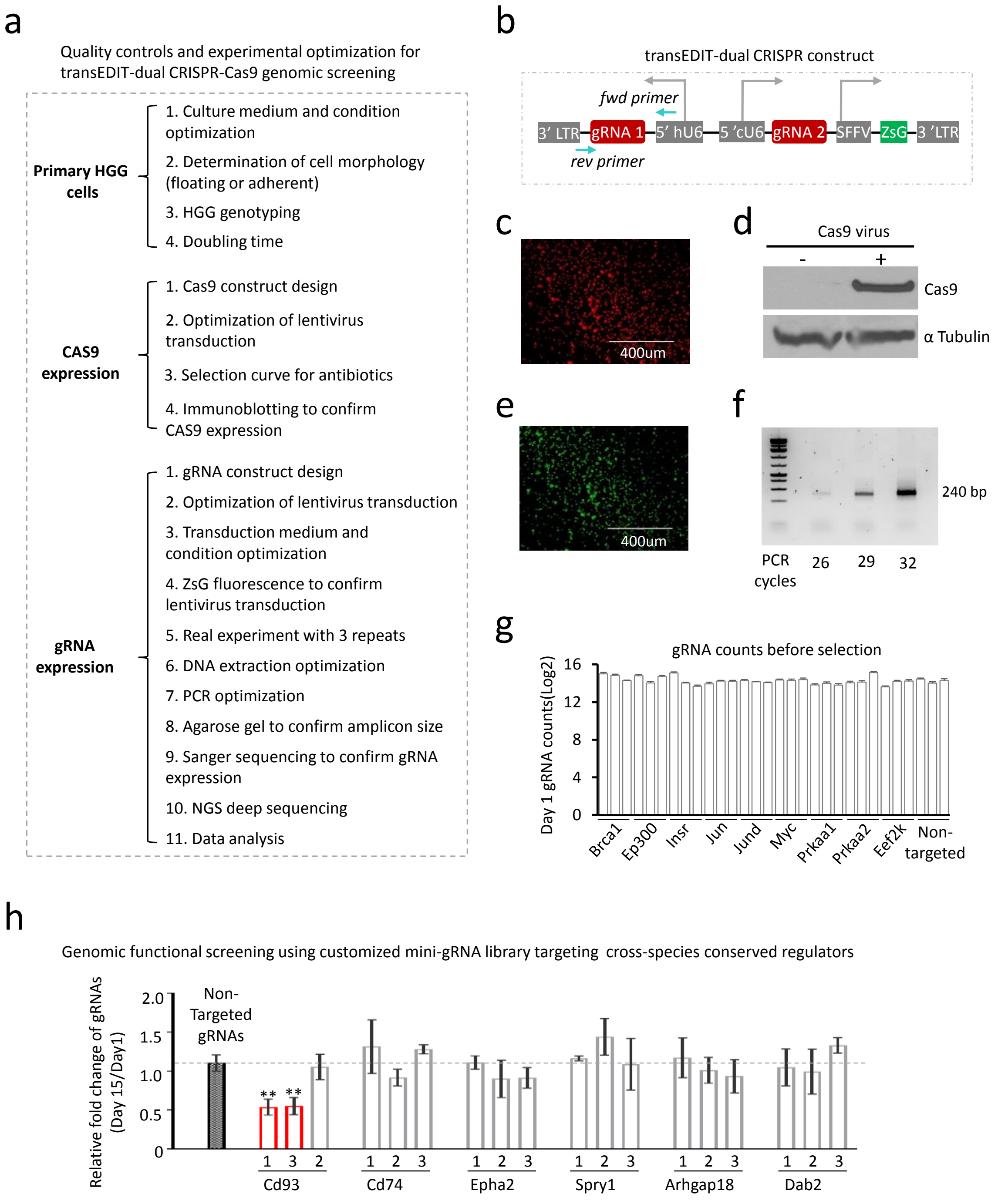
a
Quality controls and experimental optimization for transEDIT-dual CRISPR-Cas9 genomic screening
Primary HGG cells
1. Culture medium and condition optimization
2. Determination of cell morphology (floating or adherent)
3. HGG genotyping
4. Doubling time
CAS9 expression
1. Cas9 construct design
2. Optimization of lentivirus transduction
3. Selection curve for antibiotics
4. Immunoblotting to confirm CAS9 expression
gRNA expression
1. gRNA construct design
2. Optimization of lentivirus transduction
3. Transduction medium and condition optimization
4. ZsG fluorescence to confirm lentivirus transduction
5. Real experiment with 3 repeats
6. DNA extraction optimization
7. PCR optimization
8. Agarose gel to confirm amplicon size
9. Sanger sequencing to confirm gRNA expression
10. NGS deep sequencing
11. Data analysis
b
transEDIT-dual CRISPR construct
fwd primer
rev primer
3' LTR
gRNA 1
5' hU6
5 'cU6
gRNA 2
SFFV
ZsG
3 'LTR
c
400um
d
Cas9 virus
-
+
Cas9
α Tubulin
e
400um
f
240 bp
PCR cycles
26
29
32
g
gRNA counts before selection
Day 1 gRNA counts(Log2)
16
12
8
4
0
Brca1
Ep300
Insr
Jun
Jund
Myc
Prkaa1
Prkaa2
Eef2k
Non-targeted
h
Genomic functional screening using customized mini-gRNA library targeting cross-species conserved regulators
Relative fold change of gRNAs (Day 15/Day1)
2.0
1.5
1.0
0.5
0
Non-Targeted gRNAs
**
**
1 3 2
Cd93
1 2 3
Cd74
1 2 3
Epha2
1 2 3
Spry1
1 2 3
Arhgap18
1 2 3
Dab2

Supplementary Figure 10 | Quality controls for CRISPR-CAS9 functional genomic screening using transEDIT-dual system

(a) Work flow and QC steps for CRISPR-Cas9 functional genomic screening using transEDIT-dual system

(b) Simplified cartoon shows transEDIT-dual CRISPR construct with primer binding sites. Two gRNAs targeting different regions of the same genes were designed in a signal CRISPR construct. Primer binding sites for PCR amplification of gRNA 1 were shown. Fwd: forward; rev: Reverse.

(c) Image of primary HGG cells show mCherry fluorescence which co-express with *TPM3-NTRK1* cancer driver gene

(d) Stable expression of Cas9 in HGG cells as validated by immunoblot assay.

(e) gRNA integration was validated by fluorescence detection of ZsG. Image shows the detection of ZsG fluorescence in the primary HGG cells 3 days of transEDIT-dual gRNA lentivirus transduction.

(f) PCR products show amplicon size and band intensities corresponding to different PCR cycle. Theoretical amplicon size is 240bp. PCR cycle 29 was used for later analysis.

(g) Close to even distribution of gRNA counts before selection. Bar plot shows the Log2 level counts of each gRNA collected at 1 day after gRNA transduction. 3 x dual gRNAs were designed for each target gene, and the CRISPR-Cas9 experiment was repeated 3 times. Error bars indicate the s.e.m of 3 experiments.

(h) CRISPR-CAS9 genomic screening targeting top candidate targets derived through cross-species data integration. Bar pot shows the log2 level gRNA relative fold changes of top cross-species conserved regulators after selection for 15 days. Dash line indicates the mean level of all non-targeted gRNA controls. The gRNA counts of cross-species conserved regulators were compared with non-targeted gRNA controls, and red bars indicate gRNA changes that show statistical significance with a Student t test P value smaller than 0.05. Two asterisks: $p < 0.01$.

Fold change distribution and Z-value transformed distribution of MS2 and MS3 based peptide quantification comparing *NTRK-d*riven HGG to Cortex using TMT labeling.

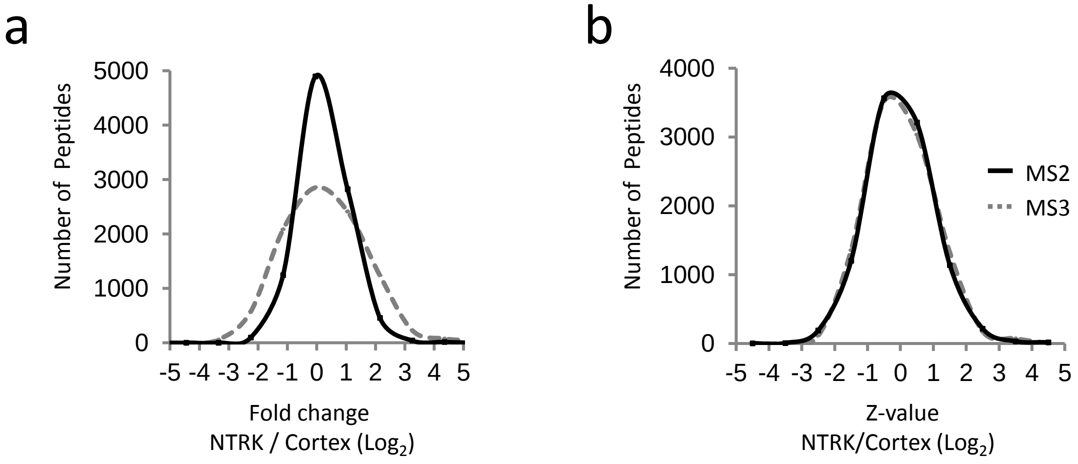

Supplementary Figure 11 | TMT-based quantification using MS2 method has essentially no impact on protein differential expression analysis after Z-scale normalization

a. Quantitative ratio compression occurred in TMT labeling strategy using MS2 compare to MS3 method. MS3 strategy can essentially eliminate ratio compression with the cost of more duty cycles and the use of low resolution MS2 data for identification, which often compromise peptide/protein identification. Comparison between MS2 and MS3 methods on the same sample using TMT labeling shows smaller difference/variance measured in MS2 method compare to MS3 method, suggesting quantitative ratio compression in MS2 analysis of TMT labeling.

b. Z scale normalization essentially eliminates the effect of ratio compression on protein differential expression analysis. Z scale transformation of the same data shows almost exact same Z value distribution in MS2 and MS3 method, suggesting similar amount of DE proteins with the same Z value cutoffs for differential expression analysis comparing MS2 and MS3 methods.
