# Supplementary material for: Deep multiomics profiling of brain tumors identifies signaling networks downstream of cancer driver genes
Source: Nat Commun. 2019 Aug 16;10:3718. doi: 10.1038/s41467-019-11661-4 (PMC6697699; doi:10.1038/s41467-019-11661-4)
Supplement: Supplementary file 3 — Description of Additional Supplementary Files [file 41467_2019_11661_MOESM3_ESM.pdf]

Description of Additional Supplementary Files

File Name: Supplementary Data 1
Description: 10-plex TMT-LC-MS quantified whole proteome of mouse specimens. Listed are coexpression clusters, gene names, protein accession (including several customized proteins of GFP, Cherry and two oncogenes), the number of peptide-spectrum matches (PSM), the number of peptides, and the summarized protein intensities from TMT reporter ions in 10 samples, the log2ratio values, and the ANOVA p values. These proteins are sorted by gene names, and then by the coexpression clusters.

File Name: Supplementary Data 2a
Description: 10-plex TMT-LC-MS quantified phosphoproteome of mouse specimens without whole proteome normalization. Listed are coexpression clusters, gene names, protein accession (including several customized proteins of GFP, Cherry and two oncogenes), phosphorylation sites, phosphosite localization scores(1-100), annotations of phosphorylation, the summarized phosphosite intensities from TMT reporter ions in 10 samples, the log2ratio values, and the ANOVA p values, The phosphosites are sorted by gene names and then by the coexpression clusters.

File Name: Supplementary Data 2b
Description: 10-plex TMT-LC-MS quantified phosphoproteome of mouse specimens after whole proteome data normalization. Listed are gene names, protein accession (including several customized proteins of GFP, Cherry and two oncogenes), phosphorylation sites, phosphosite localization scores (1-100), and the summarized phosphosite intensities from TMT reporter ions in 10 samples after normalization by the whole proteome TMT intensity. The phosphosites are sorted by gene names.

File Name: Supplementary Data 3
Description: RNAseq results of mouse specimens. Listed are gene names, FPKM value for each sample, the log2ratio values, and the B.H. adjusted ANOVA p values. Two batches of mouse specimens are analyzed. Genes with FPKM < 1 were converted to 1. Data are sorted by gene names. The genes are sorted by gene names.

File Name: Supplementary Data 4a
Description: Pathways and networks detected in all co-expression clusters. Listed include coexpression clusters, networkgroups, pathwayTerms , network Grouping PValue Corrected with Benjamini-Hochberg, pathway enrichment PValue Corrected with Benjamini-Hochberg, DE genes, % De Genes, number of DE, ontology sources and GOID. NetworkGroups with >= 5 members are accepted as functional network modules (39 WP-C modules and 28 PP-C modules). These pathways and networks are sorted by NetworkGroups and then by the coexpression clusters.

File Name: Supplementary Data 4b
Description: Full list of pathways altered in all coexpression clusters.

File Name: Supplementary Data 4c
Description: Regulatory families of proteins at whole protein level. Listed include protein types (kinases, epigenetic genes, and cancer genes), gene names, protein accessions, the log2ratio values.

File Name: Supplementary Data 4d
Description: Regulatory families of proteins at phosphoprotein level. Listed include protein types (kinases, epigenetic genes, and cancer genes), gene names, protein accessions and phosphorylation sites, the log2ratio values. Phosphosites with the largest magnitude of change are selected as representatives of phosphoprotein change.

File Name: Supplementary Data 4e
Description: 187 kinase activities are estimated by a machine learning algorithm (IKAP) using known kinase substrates from the PhosphoSitePlus database. A cutoff of 0.2 SD is applied to filter kinase activity estimations that failed to converge into a stable solution. Kinase passed the filter are listed below. Kinases with B.H. adjusted ANOVA p value > 0.05 (n = 62 survived) and with quantified substrates less than 3 (n = 47 survived) were excluded for figure 4a. For each kinase, we have included the following information: kinase names, the estimated kinase activities (ranging from -15 to 15 in the IKAP program) in 3 sample groups, the derived ANOVA P values, Benjamini–Hochberg false discovery rate (BH FDR). The list is sorted by the P values.

File Name: Supplementary Data 4f
Description: Summary of AKT substrates.

File Name: Supplementary Data 4g
Description: DE genes in PI3K-AKT pathway. Listed include gene names, phosphorylation sites, phosphorylation annotations the log2ratio values. Phosphosites with the largest magnitude of change are selected as representatives to draft figure 7a graph.
Phosphorylation annotations are functional annotations of phosphorylation based on PhosphositePlus database (08/05/2015).

File Name: Supplementary Data 4h
Description: PI3K-AKT pathway activity measurement.

File Name: Supplementary Data 4i
Description: List of pooled mini-gRNAs library targeting prioritized master regulator genes derived from the multi-Omics integrative analyses.
