# Supplementary material for: Deep multiomics profiling of brain tumors identifies signaling networks downstream of cancer driver genes
Source: Nat Commun. 2019 Aug 16;10:3718. doi: 10.1038/s41467-019-11661-4 (PMC6697699; doi:10.1038/s41467-019-11661-4)
Supplement: Supplementary file 8 — Reporting Summary [file 41467_2019_11661_MOESM8_ESM.pdf]

nature research

Corresponding author(s): Junmin Peng & Suzanne Baker

Last updated by author(s): July/10/19

Reporting Summary

Nature Research wishes to improve the reproducibility of the work that we publish. This form provides structure for consistency and transparency in reporting. For further information on Nature Research policies, see Authors & Referees and the Editorial Policy Checklist.

Statistics

For all statistical analyses, confirm that the following items are present in the figure legend, table legend, main text, or Methods section.

n/a	Confirmed	
☐	☒	The exact sample size (*n*) for each experimental group/condition, given as a discrete number and unit of measurement
☐	☒	A statement on whether measurements were taken from distinct samples or whether the same sample was measured repeatedly
☐	☒	The statistical test(s) used AND whether they are one- or two-sided<br>*Only common tests should be described solely by name; describe more complex techniques in the Methods section.*
☐	☒	A description of all covariates tested
☐	☒	A description of any assumptions or corrections, such as tests of normality and adjustment for multiple comparisons
☐	☒	A full description of the statistical parameters including central tendency (e.g. means) or other basic estimates (e.g. regression coefficient) AND variation (e.g. standard deviation) or associated estimates of uncertainty (e.g. confidence intervals)
☐	☒	For null hypothesis testing, the test statistic (e.g. *F*, *t*, *r*) with confidence intervals, effect sizes, degrees of freedom and *P* value noted<br>*Give P values as exact values whenever suitable.*
☒	☐	For Bayesian analysis, information on the choice of priors and Markov chain Monte Carlo settings
☒	☐	For hierarchical and complex designs, identification of the appropriate level for tests and full reporting of outcomes
☐	☒	Estimates of effect sizes (e.g. Cohen's *d*, Pearson's *r*), indicating how they were calculated

Our web collection on statistics for biologists contains articles on many of the points above.

Software and code

Policy information about availability of computer code

Data collection	Thermo Xcalibur V3.1
Data analysis	JUMP software V1.13.0; WGCNA (Langfelder P. 2008); ClueGO(Bindea G. 2009); IKAP (Mischnik M.2016)

For manuscripts utilizing custom algorithms or software that are central to the research but not yet described in published literature, software must be made available to editors/reviewers. We strongly encourage code deposition in a community repository (e.g. GitHub). See the Nature Research guidelines for submitting code & software for further information.

Data

Policy information about availability of data

All manuscripts must include a data availability statement. This statement should provide the following information, where applicable:

- Accession codes, unique identifiers, or web links for publicly available datasets
- A list of figures that have associated raw data
- A description of any restrictions on data availability

Raw genomic and proteomic data that support the findings of this study have been deposited in GEO with an accession number: GSE114331 [https://www.ncbi.nlm.nih.gov/geo/query/acc.cgi?acc=GSE114331], and ProteomeXchange with an accession number: PXD005360 [http://proteomecentral.proteomexchange.org/cgi/GetDataset?ID=PXD005360]. Analyzed data are included in this published article and its supplementary files. Source data underlying Figs 1b, 1d, 2a-e, 3b-f, 4a-d, 5b, 5c, 6a-d, 7a-f, and 8b are provided as a Source Data file.

Field-specific reporting

Please select the one below that is the best fit for your research. If you are not sure, read the appropriate sections before making your selection.

☒ Life sciences ☐ Behavioural & social sciences ☐ Ecological, evolutionary & environmental sciences

For a reference copy of the document with all sections, see nature.com/documents/nr-reporting-summary-flat.pdf

Life sciences study design

All studies must disclose on these points even when the disclosure is negative.

Sample size	No sample-size calculations were performed. Sample size was determined by the maximal number of TMT multiplexing labeling reagents available
Data exclusions	No data exclusions applied
Replication	Replicate experiments were successful
Randomization	No randomization of mouse are applied. Mice analyzed were age and sex matched whenever possible.
Blinding	No blinding applied, Data reported for mouse tumor analyses are not subjective but rather based on global proteome quantification.

Reporting for specific materials, systems and methods

We require information from authors about some types of materials, experimental systems and methods used in many studies. Here, indicate whether each material, system or method listed is relevant to your study. If you are not sure if a list item applies to your research, read the appropriate section before selecting a response.

Materials & experimental systems

n/a	Involved in the study	
☐	☒	Antibodies
☒	☐	Eukaryotic cell lines
☒	☐	Palaeontology
☐	☒	Animals and other organisms
☒	☐	Human research participants
☒	☐	Clinical data

Methods

n/a	Involved in the study	
☒	☐	ChIP-seq
☒	☐	Flow cytometry
☒	☐	MRI-based neuroimaging

Antibodies

Antibodies used	p-C-Myc (Abcam, 32029); Tubulin and PDGFRA (Santa Cruz, 23948, and 338); EphA2, pEphA2, and C-Myc (Cell Signaling, 6997, 6347, and 9402)
Validation	Validation on antibodies are available on manufacturer's websites

Animals and other organisms

Policy information about studies involving animals; ARRIVE guidelines recommended for reporting animal research

Laboratory animals	Animal information were provided in method section on in the manuscript
Wild animals	No wild animal was applied in this study
Field-collected samples	No field-collected sample was applied in this study
Ethics oversight	Mouse experiments were approved by Institutional Animal Care and Use Committee and are in compliance with national and institutional guidelines

Note that full information on the approval of the study protocol must also be provided in the manuscript.
